# Mapping Health-Related Quality of Life, Anxiety, and Depression in Patients with Head and Neck Cancer Diagnosed with Malnutrition Defined by GLIM

**DOI:** 10.3390/nu13041167

**Published:** 2021-04-01

**Authors:** Ylva Tiblom Ehrsson, Per Fransson, Sandra Einarsson

**Affiliations:** 1 Department of Surgical Sciences, Section of Otorhinolaryngology and Head & Neck Surgery, Uppsala University, SE-751 85 Uppsala, Sweden; 2 Department of Nursing, Umeå University, SE-901 87 Umeå, Sweden; per.m.fransson@umu.se; 3 Department of Food, Nutrition and Culinary Science, Umeå University, SE-901 87 Umeå, Sweden; sandra.einarsson@umu.se

**Keywords:** head and neck cancer, malnutrition, EORTC QLQ-H&N35, HADS, weight loss, CRP

## Abstract

Patients with cancer deal with problems related to physical, psychological, social, and emotional functions. The aim was to investigate malnutrition defined by the Global Leadership Initiative on Malnutrition (GLIM) criteria in relation to health-related quality of life, anxiety, and depression in patients with head and neck cancer. This was a prospective observational research study with 273 patients followed at the start of treatment, seven weeks, and one year. Data collection included nutritional status and support, and the questionnaires: European Organization for Research and Treatment of Cancer Head and neck cancer module (EORTC QLQ-H&N35) and the Hospital Anxiety and Depression Scale (HADS). Malnutrition was defined using the GLIM criteria. The study showed that patients with malnutrition had significantly greater deterioration in their health-related quality of life at seven weeks. On a group level, health-related quality of life was most severe at this time point and some scores still implied problems at one year. Significantly, more patients reported anxiety at the start of treatment whereas significantly more patients reported depression at seven weeks. Over the trajectory of care, the need for support often varies. Psychosocial support is imperative and at the end of treatment extra focus should be put on nutritional interventions and managing treatment-related symptoms to improve nutritional status and health-related quality of life. In the long-term, head and neck cancer survivors need help to find strategies to cope with the remaining sequel.

## 1. Introduction

Over the years, previous research has shown that patients with cancer not only have to deal with impaired physical function related to the disease and treatment, but also with challenges from a psychological, social, and emotional perspective. In patients with head and neck cancer (HNC), the tumor itself and treatment-related toxicities may lead to aesthetic alterations and dysfunctions e.g., of the oral cavity and the swallowing procedure, and speech impairment [1,2], which can lead to health-related quality of life (HRQoL) deterioration and emotional distress with depression and anxiety [3]. 

HNC refers to a heterogeneous group of cancer in the upper aero digestive tract and is a collective term for nine different diagnoses [1]. The main treatment is external beam radiotherapy (RT) delivered either as a single treatment or combined with surgery and/or pharmacological therapy (chemotherapy or antibody therapy). One of the most reported problems by patients with HNC is treatment-related nutritional problems [4], i.e., smell and taste alterations, xerostomia, mucositis, dysphagia, chewing problems, pain in the oral cavity and throat, and trismus [1,5,6,7,8]. Eating problems lead to deterioration in nutritional status and a nadir of weight loss has been reported in earlier studies to occur around six months after treatment [9,10]. A study by Petruson et al. [11] on 49 patients with HNC showed that patients with weight loss of ≥10% during six months (a set period between three months before and three months after diagnosis) had poorer HRQoL at diagnosis compared to those with less weight loss. Hence, problems related to a poor nutritional status affect the patients in many ways and have negative impacts on physical, psychological, existential, as well as social wellbeing [12,13] and may lead to reduced HRQoL and increased mortality [14].

A worldwide consensus to define malnutrition has been absent up until now. In 2018, experts from several international organizations agreed on a definition for malnutrition in adults, i.e., the Global Leadership Initiative on Malnutrition (GLIM) [15]. They suggest diagnosing malnutrition by combining at least one phenotypic criterion (body weight loss, low body mass index [BMI], or reduced muscle mass) with one etiologic criterion (reduced food intake/assimilation or inflammation). In 2020, we examined the prevalence of malnutrition according to GLIM in HNC [16], and in our most recent publication [17], we further examined GLIM in relation to different patient-, tumor-, and treatment-related factors. To continue to build an evidence base for GLIM, for this study, we wanted to understand the relationship between malnutrition according to GLIM with HRQoL and emotional distress on larger groups of patients. 

The aim with the present study was to investigate malnutrition defined by the GLIM criteria in relation to health-related quality of life, anxiety, and depression in patients with head and neck cancer at the start of treatment and up to one year after treatment.

## 2. Materials and Methods

This is the third study from this research group about GLIM [16,17], which derives from a larger Swedish prospective observational research study (ClinicalTrials.gov NCT03343236). 

### 2.1. Subjects

Patients with untreated and curative intent HNC with a performance status of 0–2 rendering from the World Health Organization (WHO) were included. Exclusion criteria were inability to understand the Swedish language, severe alcohol abuse, cognitive diagnoses such as senile dementia or mental disturbance, or malignant neoplasm previously treated within the past five years. The patients were recruited from October 2015 to July 2019 at three tertiary referral hospitals and the follow-ups took place there or at the local hospital. During that period, 288 patients accepted participation, and 273 patients were included in the present study. The patients that were excluded had missing data at the first follow-up at seven weeks after the start of treatment due to palliative care or being deceased (*n* = 4), dropouts at seven weeks (*n* = 1), or missing values of GLIM (*n* = 10). Of the included 273 patients, 31 did not complete the last follow-up at one year. This was due to residual disease (*n* = 3), deceased patients (*n* = 22), or dropouts (*n* = 6). 

### 2.2. Data Collection 

Data for this study was collected at the initiation of treatment, with follow-ups at seven weeks after the start of treatment, and one year after the end of treatment. For patients treated with RT, the follow-up at seven weeks after the start of treatment corresponded to the end of RT. A database for the research study has been developed to maintain easy, reliable, and safe data collection (data.dynareg.se). Data extracted from the database for this study was: background data to present patient (age, sex, living arrangements, working situation, and smoking habits), tumor (tumor location, tumor stage), and treatment characteristics (external radiotherapy, surgery, pharmacological therapy, brachytherapy); and nutritional assessment parameters. In addition, a blood sample for C-reactive protein (CRP) was collected and the analysis of CRP was carried out in certified laboratories.

### 2.3. Nutritional Assessment

Nutritional status was continuously monitored during the patients’ cancer treatment, and according to local guidelines they were offered nutritional support when they had a body weight loss of >5%, problems with oral eating or expected severe nutritional problems due to advanced tumor of stage IV. Percentage body weight loss was calculated at seven weeks after the start of treatment and one year after the end of treatment, with weight at the start of treatment as the reference weight. Clinically relevant weight loss was defined as >5% within six months or >10% beyond six months, respectively [15]. Cut-offs for BMI were: underweight <20; normal weight 20–24.99; and overweight/obesity ≥25 if <70 years [15]. For patients over 70 years, BMI <22 was considered underweight and BMI between 22 and 27 was considered normal. The patient’s fat-free mass (FFM) was measured using an 8-electrode bioelectrical impedance analysis (BIA) device (type BC-418MA, Tanita Corporation, Tokyo, Japan). The patients were also asked if they could maintain oral intake or needed nutritional support (total or partial use of tube feeding/parenteral nutrition).

The patients in this study were not screened for malnutrition as all patients with HNC are at potential risk for malnutrition due to the location of the tumor and the given treatment. Patients were diagnosed with malnutrition at seven weeks after the start of treatment if having one combination of GLIM present i.e., one phenotypic criterion and one etiologic criterion. The choice of time point relies on our previous study showing the highest frequency of malnutrition at seven weeks [15]. The phenotypic GLIM criteria were defined as >5% body weight loss within six months, BMI <20 or <22 if >70 years, or fat-free mass index (FFMI) <15 FFM/m^2^ for women and <17 FFM/m^2^ for men [15]. Since the BIA equipment was only available at the three tertiary referral hospitals, patients assessed at their local hospital at the follow-ups had no value for FFMI (*n* = 107). The etiologic GLIM criteria were defined as partial or no food intake with the need for nutritional support or CRP >5 mg/L as suggested by the European Society for Clinical Nutrition and Metabolism, ESPEN [18]. 

### 2.4. Health-Related Quality of Life, Anxiety, and Depression

Patients were asked to answer two questionnaires about HRQoL and emotional distress. The patients were able to fill in a digital version of the questionnaires where data were transferred straight into the database (data.dynareg.se), or they were able to fill in a paper version. Research nurses transferred the results from the paper version into the database. The number of patients answering each question at the start of treatment and the two follow-ups is presented in Table 2 and Appendix A.

The HRQoL questionnaire used was the European Organization for Research and Treatment of Cancer Head and neck cancer module (EORTC QLQ-H&N35) [19], which consists of 35 questions related to problems caused by the tumor location and the given treatment. It includes seven multi-item scales regarding: pain, swallowing, senses problems, speech problems, trouble with social eating and social contact, and less sexuality, and additionally, eleven single items. Patients respond on a 4-point Likert scale from 1 = not at all to 4 = very much, except for five questions which are rated on a yes-or-no scale. The results from the EORTC QLQ-H&N35 module should be interpreted such that a higher score indicates a worse problem. For the five questions rated on a yes-or-no scale, the scores indicate the percentage of yes answers. Clinically relevant changes in EORTC QLQ-H&N35 scores were defined as a ≥10-point change from baseline [20].

The Hospital Anxiety and Depression Scale (HADS) [21] was used to screen for psychiatric morbidity/emotional distress and is a valid and reliable self-rating screening tool. It consists of 14-item questions, seven for anxiety, and seven for depression. The scores grade from 0–3 (4-point Likert scale) and they are separately summarized from 0 to 21. Cut-off scores are 0–6 normal, 7–10 mild to moderate, and >10 severe.

### 2.5. Statistical Analysis

Descriptive statistical methods are presented for continuous variables as mean ± standard deviation (SD), and categorical variables are presented as numbers (%). The outcome binary variable is malnutrition defined by GLIM (yes/no) at seven weeks after the start of treatment. Differences in background variables between patients with and without malnutrition were measured using the Pearson’s chi-squared test or the Independent Samples *T*-test. HRQoL and anxiety/depression between patients with and without malnutrition were measured using the Mann–Whitney U-test. Wilcoxon Signed Rank Test was used to analyze the statistically significant change in the distribution of patients to different nutritional parameters (BMI and use of nutritional support) and different scores in HADS from start of treatment to the two follow-ups, respectively. Descriptive statistics were used to present numbers (%) of patients with clinically relevant changes in HRQoL (10 points higher compared to the score reported at the start of treatment) for symptoms found statistically significant between patients with and without malnutrition. A p value of < 0.05 was considered statistically significant and all tests were two-tailed. For all statistical analyzes, IBM SPSS statistics version 27 (IBM, Armonk, NY, USA) was used.

## 3. Results

The characteristics of the 273 patients at the start of treatment are presented in Table 1. The mean age was 63 years (±11 years), and the male-to-female ratio was 2.64:1 (198 males, 75 females). Most patients had tumor of the oropharynx 124/273 (45.4%) and 160/273 (58.6%) had stage I-II cancer.

Table 1 also display patient-, tumor-, and treatment characteristics in patients diagnosed with malnutrition according to GLIM (*n* = 123) vs. patients without malnutrition (*n* = 150) at seven weeks after the start of treatment. Statistically significant differences between the groups were seen for tumor site (*p* = 0.041), tumor stage (*p* < 0.001), and treatment type (*p* < 0.001).

### 3.1. Nutritional Status and Nutritional Support

**Figure 1:**
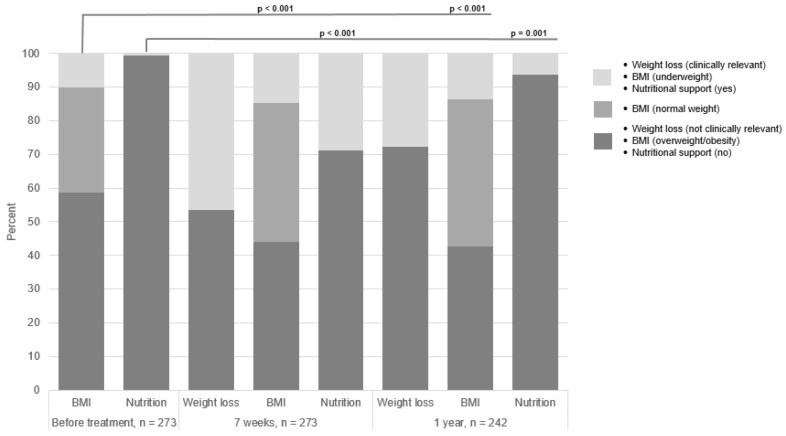
Nutritional status and use of nutritional support presented with percent (%) over time, i.e., start of treatment (*n* = 273), seven weeks after the start of treatment (*n* = 273), and one year after the end of treatment (*n* = 242) in patients with head and neck cancer. The Wilcoxon Signed Rank Test was used to analyze the statistically significant change in the distribution of patients to different nutritional parameters from start of treatment to the two follow-ups, respectively.

According to BMI, few patients were underweight at the start of treatment and only two patients needed nutritional support (Figure 1). At seven weeks after the start of treatment, nearly half of the patients displayed a clinically relevant weight loss 127/273 (46.5%). There was a statistically significant change in the distribution of patients to different BMI groups (*p* < 0.001) and use of nutritional support (*p* < 0.001) at seven weeks compared to the start of treatment. The same was also shown for the follow-up at one year: BMI (*p* < 0.001) and use of nutritional support (*p* = 0.001).

### 3.2. Malnutrition in Relation to Health-Related Quality of Life, Anxiety, and Depression

#### 3.2.1. Start of Treatment

At the start of treatment, patients with malnutrition according to GLIM scored worse in pain (*p* = 0.003), swallowing (*p* < 0.001), senses problems (*p* = 0.013), trouble with social eating (*p* < 0.001), pain killers (*p* = 0.023), nutritional supplements (*p* = 0.002), and weight loss (*p* = 0.001) compared to patients with no malnutrition (Appendix A). No significant differences were seen between the two groups for any of the other symptoms or anxiety (*p* = 0.889) or depression (*p* = 0.948).

#### 3.2.2. Seven Weeks after Start of Treatment

Seven weeks after the start of treatment displayed the highest mean values in HRQoL (i.e., worsening in symptoms), and patients with malnutrition according to GLIM scored worse in many of the symptoms compared to patients with no malnutrition (Table 2). Patients with malnutrition scored significantly worse in depression compared to patients with no malnutrition (*p* = 0.047). No significant difference was seen between the two groups for anxiety (*p* = 0.290). 

For the symptoms showing statistical significance between the two groups, clinically relevant deteriorations, i.e., patients scoring at least 10 points higher at seven weeks after the start of treatment compared to the score reported at the start of treatment, are shown in Table 3. The most clinically relevant symptom for patients with malnutrition according to GLIM was sticky saliva 93/104 (89.4%). The corresponding number for patients without malnutrition was 97/137 (70.8%). 

#### 3.2.3. One Year after End of Treatment

At one year after the end of treatment, patients with malnutrition according to GLIM scored worse in pain (*p* = 0.009), swallowing (*p* < 0.001), trouble with social eating (*p* = 0.007), dry mouth (*p* = 0.005), and sticky saliva (*p* = 0.017) (Appendix A). No significant differences were seen between the two groups for any of the other symptoms or anxiety (*p* = 0.872) or depression (*p* = 0.489).

Clinically relevant deteriorations, i.e., patients scoring at least 10 points higher in HRQoL at one year after the termination of treatment compared to the score reported at the start of treatment, are shown in Appendix B. The most clinically relevant symptom for patients with malnutrition according to GLIM was dry mouth 56/80 (70.0%). The corresponding number for patients without malnutrition was 49/97 (50.5%).

### 3.3. Anxiety and Depression over Time

Most patients reported moderate 56/260 (21.5%) or severe 22/260 (8.5%) anxiety at the start of treatment compared with the two follow-ups at seven weeks after the start of treatment and one year after the end of treatment (Table 4). There was a statistically significant decrease in anxiety from the start of treatment to seven weeks after the start of treatment (*p* = 0.031). However, most patients reported moderate 40/234 (17.1%) or severe 18/234 (7.7%) depression at seven weeks after the start of treatment with a statistically significant increase from the start of treatment (*p* < 0.001).

## 4. Discussion

In this prospective observational research study, we mapped HRQoL, anxiety, and depression in patients with HNC diagnosed with malnutrition defined by GLIM. The main findings were that patients with malnutrition had significantly greater deterioration in their HRQoL at seven weeks. On a group level, HRQoL was most severe at this time point and some scores still implied problems at one year. The present study is, to our knowledge, the first using GLIM to assess nutritional status in relation to HRQoL, anxiety, and depression in patients with HNC. This new information can enable healthcare professionals to give better support to patients from the start of HNC treatment, as well as in a longer perspective.

The most common and greatest nutritional problems in HNC are usually caused by the side effects from the given treatment that have effects on HRQoL including nutritional issues. Patients with malnutrition according to GLIM showed greater deterioration in their HRQoL according to EORTC QLQ-H&N35 at seven weeks after the start of treatment compared to patients without malnutrition. For the group in total, the mean values of HRQoL scores were in general most severe at this time point. For patients treated with RT, this is the time point when the systematic daily support from the healthcare system during treatment ends, i.e., at a time point when nutritional status [16,17] and HRQoL [22,23] often are at their worse. This indicates the importance of extra support to the patients when returning home after treatment. A study by Isenring et al. [24] showed, for example, that it is possible to improve the deterioration found in nutritional status and HRQoL during treatment with early and intensive nutritional interventions. 

One item from the EORTC QLQ-H&N35 questionnaire that needs to be given extra attention is “sticky saliva”. Patients with malnutrition according to GLIM scored significantly worse on that item at seven weeks after the start of treatment compared to patients without malnutrition. The majority of patients with malnutrition 93/104 (89.4%) had at least a ten-point higher score compared to the score reported at the start of treatment, indicating a clinically relevant deterioration of that symptom [20]. Changes in the quantity and composition of saliva are common acute and late complications of HNC treatment [1,5,6]. A prospective cohort study by Likhterov et al. [25] on 582 patients with HNC assessed stimulated saliva weight from treatment start up to three years post-treatment. They showed that the post-treatment saliva weight was significantly lower compared to before treatment. It is evident that this treatment sequela is common, and that extra effort should be put into helping patients manage changes in the quantity and composition of saliva to be able to improve nutritional status and HRQoL in HNC survivors.

Patients with malnutrition according to GLIM scored significantly worse for “trouble with social eating” at seven weeks after the start of treatment compared to patients without malnutrition, and the majority 83/98 (84.7%) had at least a ten-point higher score compared to the score reported at the start of treatment. Treatment-related nutritional problems are one of the most commonly reported problems by patients with HNC [4] and earlier studies have shown that these may affect social aspects related to food and eating [13,26,27]. Not being able to eat in a “socially desirable way” leads many patients to refrain from eating with others [13,27] and rehabilitation has been shown to significantly improve patient reported “trouble with social eating” [28]. Hence, adopting a holistic approach, i.e., enabling support to all aspects of food and eating for HNC survivors is important not only to recover the patients’ nutritional status, but also to improve HRQoL.

At one year after the termination of treatment, some scores of the EORTC QLQ-H&N35 questionnaire still implied extensive problems. Patients with malnutrition according to GLIM at seven weeks after the start of treatment scored significantly worse in, for example, “swallowing” and “dry mouth”, whereas 56/80 (70.0%) of patients with malnutrition had a clinical deterioration in “dry mouth” at one year compared to pre-treatment values. The late effects of HNC treatment and that some treatment sequelae may even become chronic [1,5,6] are well known. One important aspect to recognize when studying HRQoL is that it may change over time and according to the patient’s coping ability [29]. Ganzer et al. [26] describe an “adaption” of the eating situation by HNC survivors. Some patients may even adapt so well that they are unable to recognize that they suffer from long-term treatment sequelae [30], i.e., the situation becomes a “new normal” [13,27]. It is therefore important to consider that improvements in HRQoL might be the result of a better physical function per se, but also the result of the patient’s ability to adapt to the new situation. Earlier studies have shown that many HNC survivors have unmet needs after treatment [31,32] and it is suggested that the rehabilitation approach in HNC survivors should be individualized and patient-focused, and given with a holistic approach [33]. Hence, the lack of proper rehabilitation strategies for HNC survivors is an issue that needs to be further addressed by the healthcare system.

Although the mean cut-off score for anxiety and depression according to HADS was within the normal range (0-6) at the start of treatment and the two follow-ups, patients with malnutrition scored significantly lower for depression at seven weeks compared to patients without malnutrition. In line with earlier studies [3,34], significantly more patients had moderate or severe anxiety at the start of treatment when compared to the follow-up at seven weeks after the start of treatment. However, significantly more patients had moderate or severe depression at the seven weeks follow-up compared to the start of treatment. Symptoms of anxiety and depression are often associated with a higher symptom burden [3], which addresses the need for tailored care that focuses on both the presence of anxiety or depression and additional symptoms. To further investigate different patient-related factors such as e.g., socio-economic status and motivation in relation to the ability to recover in nutritional status after HNC treatment would be an interesting approach for future studies.

The main strength of the study is the consecutive follow-up of patients over time, the large sample size, and the assessment of all GLIM criteria. The EORTC QLQ-H&N35 [19] and HADS [21] are well recognized and validated tools used for quantitative measures of HRQoL, anxiety, and depression. A limitation of the study is the high number of statistical tests that pose a risk of type 1 errors. Other limitations could be the number of missing values for FFMI as well as the rather large dropouts (about 30%) of patients not answering EORTC QLQ-H&N35 and HADS at the one-year follow-up. When interpreting the results, the reader should be aware that patients with HNC are always at risk for malnutrition due to the tumor location and the treatment given [35]. Patients with malnutrition more often had tumors of the oropharynx and stage III-IV cancer, and were more often treated with chemoradiotherapy with or without surgery. Hence, this issue may have influenced the result. Also, since the GLIM criteria are very new, more work is needed to test its validity as well as its clinical applicability and feasibility. One could also argue that nutritional screening should be done pre-GLIM, but patients with HNC are always at risk for malnutrition so consequently all patients were included.

## 5. Conclusions

Patients with HNC need support that may vary in intensity and form over the trajectory of care. In relation to the treatment period, psychosocial support is imperative to help patients who suffer from anxiety and depression. Nutritional intervention needs to be addressed from the start and throughout the trajectory of care. At the end of treatment, an extra focus should be put on nutritional interventions and managing treatment-related symptoms to improve nutritional status and HRQoL. In a long-term perspective after the termination of treatment, HNC survivors need help to find strategies to cope with the remaining treatment sequelae. Rehabilitation strategies for HNC survivors are an issue that needs to be addressed by the healthcare system by adopting individualized and patient-focused follow-up routines given with a holistic approach.

## Figures and Tables

**Table 1:** Characteristics of the studied patients with head and neck cancer regarding the group in total (*n* = 273), patients with malnutrition (*n* = 123) at seven weeks after the start of treatment defined by the Global Leadership Initiative on Malnutrition (GLIM), and patients without malnutrition (*n* = 150).

Characteristics	Sub-Groups	Total	Malnutrition		*p* Value *
			Yes	No	
**Age (years), mean (SD)**		63.0 (11.0)	63.7 (10.6)	62.5 (11.4)	0.371
**Age, n (%)**	<70 years	191 (70.0)	85 (69.1)	106 (70.7)	0.780
	≥70 years	82 (30.0)	38 (30.9)	44 (29.3)	
**Gender, n (%)**	Female	75 (27.5)	36 (29.3)	39 (26.0)	0.547
	Male	198 (72.5)	87 (70.7)	111 (74.0)	
**Working situation, n (%)**	Currently working	142 (52.0)	58 (47.2)	84 (56.0)	0.146
	Unemployed, on sick leave, pensioner	131 (48.0)	65 (52.8)	66 (44.0)	
**Smoking, n (%)**	Never smoked	91 (33.3)	40 (32.5)	51 (34.0)	0.796
	Ex-/smoker	182 (66.7)	83 (67.5)	99 (66.0)	
**Living arrangements, n (%)**	Living with someone	204 (74.7)	91 (74.0)	113 (75.8)	0.725
	Living alone	68 (24.9)	32 (26.0)	36 (24.2)	
	Missing	1 (0.4)	-	-	
**Tumor site, n (%)**	Oropharynx	124 (45.4)	62 (50.4)	62 (41.3)	**0.041**
	Oral cavity	75 (27.5)	37 (30.1)	38 (25.3)	
	Larynx	30 (11.0)	7 (5.7)	23 (15.3)	
	Other ^†^	44 (16.1)	17 (13.8)	27 (18.0)	
**Tumor stage ^‡^, n (%)**	I-II	160 (58.6)	51 (41.5)	109 (73.6)	**<0.001**
	III-IV	111 (40.7)	72 (58.5)	39 (26.4)	
	Not applicable	2 (0.7)	-	-	
**Treatment type, n (%)**	RT ^§^ ± surgery	149 (54.6)	57 (46.3)	92 (61.3)	**<0.001**
	Surgery	24 (8.8)	2 (1.6)	22 (14.7)	
	Chemoradiotherapy ± surgery	75 (27.5)	50 (40.7)	25 (16.7)	
	RT ^§^ ± surgery + other pharmacological treatment	16 (5.9)	9 (7.3)	7 (4.7)	
	Brachytherapy	9 (3.3)	5 (4.1)	4 (2.7)	

* Malnutrition according to GLIM vs. no malnutrition using the Independent Samples T test or the Pearson’s chi-squared test. Statistically significant *p*-values (<0.05) are shown in bold text. † Hypopharynx, nasopharynx, salivary gland cancer, nasal and sinus cancer, cancer of the external auditory canal, ear cancer, and cancer of unknown primary. ‡ The Union for International Cancer Control’s (UICC) 8. § External radiotherapy.

**Table 2:** Numbers (N), mean, and standard deviation (SD) on HADS and EORTC QLQ-H&N35 at seven weeks after the start of treatment for head and neck cancer regarding the total group, patients with malnutrition defined by the Global Leadership Initiative on Malnutrition (GLIM), and patients without malnutrition.

	Total			Malnutrition			No malnutrition			
	*N*	Mean	SD	*N*	Mean	SD	*N*	Mean	SD	*p* Value *
**HADS ^†^**										
Total score anxiety	245	3.69	3.83	108	3.91	3.75	137	3.51	3.90	0.290
Total score depression	242	3.88	3.77	108	4.51	4.15	137	3.40	3.38	**0.047**
**EORTC QLQ-H&N35 ^‡^**										
Pain	248	46.00	26.84	109	56.98	25.26	139	37.39	24.90	**<0.001**
Swallowing	248	40.52	30.00	109	55.99	27.41	139	28.40	26.14	**<0.001**
Senses problems	248	45.56	28.74	109	54.89	29.34	139	38.25	26.12	**<0.001**
Speech problems	238	30.72	27.03	105	38.41	28.75	133	24.64	24.02	**<0.001**
Trouble with social eating	237	42.92	26.11	104	55.05	25.64	133	33.44	22.36	**<0.001**
Trouble with social contact	236	12.08	18.03	104	15.54	21.13	132	9.34	14.66	**0.028**
Less sexuality	217	50.38	38.24	97	62.54	35.7	120	40.56	37.53	**<0.001**
Teeth	247	14.30	24.46	108	14.51	24.66	139	14.15	24.40	0.831
Opening mouth	248	28.09	31.09	109	33.94	31.09	139	23.50	30.42	**0.003**
Dry mouth	248	58.33	34.88	109	62.08	35.0	139	55.40	34.65	0.114
Sticky saliva	248	68.82	33.73	109	82.57	26.3	139	58.03	35.06	**<0.001**
Coughing	248	40.19	33.44	109	51.07	34.4	139	31.65	30.12	**<0.001**
Feeling ill	248	33.47	31.24	109	43.73	33.2	139	25.42	27.09	**<0.001**
*Pain killers*	238	78.57	41.12	105	82.86	37.87	133	75.19	43.36	0.153
*Nutritional supplements*	238	68.07	46.72	105	74.29	43.92	133	63.16	48.42	0.068
*Feeding tube*	237	24.47	43.08	105	45.71	55.06	132	7.58	26.56	**<0.001**
*Weight loss*	235	69.79	46.02	105	86.67	34.16	130	56.15	49.81	**<0.001**
*Weight gain*	235	15.74	36.50	104	11.54	32.10	131	19.08	39.48	0.115

* Malnutrition according to GLIM vs. no malnutrition using the Mann–Whitney U-test. Statistically significant *p*-values (<0.05) are shown in bold text. † HADS Hospital Anxiety and Depression Scale. ‡ EORTC QLQ-H&N35 European Organization for Research and Treatment of Cancer Quality of Life Questionnaire-Head and Neck 35. Higher scores indicate more severe symptoms. For parameters in italics, the scores indicate the percentage of yes answers.

**Table 3:** Clinically relevant deterioration from the start of treatment to seven weeks the after start of treatment in a number of EORTC QLQ-H&N35 scales proven to be significantly different between patients with malnutrition defined by the Global Leadership Initiative on Malnutrition (GLIM) and patients without malnutrition.

	Malnutrition			No malnutrition		
	n	S ≥ 10 ^†^	%	n	S ≥ 10 ^†^	%
Sticky saliva	104	93	89.4	137	97	70.8
Trouble with social eating	98	83	84.7	128	81	63.3
Senses problems	104	86	82.7	137	104	75.9
Swallowing	104	81	77.9	136	72	52.9
Less sexuality	88	65	73.9	114	51	44.7
Pain	104	75	72.1	137	87	63.5
Speech problems	100	72	72.0	129	68	52.7
Coughing	104	64	61.5	137	56	40.9
Feeling ill	104	64	61.5	136	48	35.3
Opening mouth	104	55	52.9	137	49	35.8
Trouble with social contact	99	36	36.4	128	26	20.3

† S ≥ 10: Number of patients reporting scores from the EORTC QLQ-H&N35 European Organization for Research and Treatment of Cancer Quality of Life Questionnaire-Head and Neck 35 at seven weeks after the start of treatment of at least 10 points higher compared to the score reported at the start of treatment, indicating a clinically relevant deterioration of the symptom.

**Table 4:** The distribution of patients to different cut-off scores for the Hospital Anxiety and Depression Scale (HADS) at the start of treatment and the two follow-ups. Data is shown in total as well as patients with malnutrition defined by the Global Leadership Initiative on Malnutrition (GLIM) and patients without malnutrition.

	Start of treatment				Seven weeks					One year				
		Mild ^†^	Moderate ^†^	Severe ^†^		Mild ^†^	Moderate ^†^	Severe ^†^			Mild ^†^	Moderate ^†^	Severe ^†^	
	n	n (%)	n (%)	n (%)	n	n (%)	n (%)	n (%)	*p* Value *	n	n (%)	n (%)	n (%)	*p* Value *
HADS anxiety, total	260	182 (70.0)	56 (21.5)	22 (8.5)	235	183 (77.9)	35 (14.9)	17 (7.2)	**0.031**	171	138 (80.7)	21 (12.3)	12 (7.0)	0.294
*- Malnutrition*	144	100 (69.4)	29 (20.1)	15 (10.4)	103	76 (73.8)	20 (19.4)	7 (6.8)		77	62 (80.5)	12 (15.6)	3 (3.9)	
*- No malnutrition*	116	82 (70.7)	27 (23.3)	7 (6.0)	132	107 (81.1)	15 (11.4)	10 (7.6)		94	76 (80.9)	9 (9.6)	9 (9.6)	
HADS depression, total	261	232 (88.9)	21 (8.0)	8 (3.1)	234	176 (75.2)	40 (17.1)	18 (7.7)	**<0.001**	175	153 (87.4)	17 (9.7)	5 (2.9)	0.290
*- Malnutrition*	116	104 (89.7)	9 (7.8)	3 (2.6)	101	69 (68.3)	21 (20.8)	11 (10.9)		78	69 (88.5)	7 (9.0)	2 (2.6)	
*- No malnutrition*	145	128 (88.3)	12 (8.3)	5 (3.4)	133	107 (80.5)	19 (14.3)	7 (5.3)		97	84 (86.6)	10 (10.3)	3 (3.1)	

* The Wilcoxon Signed Rank Test was used to analyze the statistically significant change in the distribution of patients to different scores in HADS at the two follow-ups compared to start of treatment. Statistically significant *p*-values (<0.05) are shown in bold text. † HADS score 0–6 = Mild; 7–10 = Moderate; >10 = Severe.

## Data Availability

The data are available to the corresponding author (Y.TE) upon reasonable request.

## Appendix A

**Table A1:** Numbers (N), mean, and standard deviation (SD) on HADS and EORTC QLQ-H&N35 at the start of treatment for head and neck cancer and one year after the end of treatment, respectively. Data is shown for the group in total, patients with malnutrition defined by the Global Leadership Initiative on Malnutrition (GLIM), and patients without malnutrition.

	Start of Treatment										One Year After End of Treatment									
	Total			Malnutrition			No malnutrition				Total			Malnutrition			No Malnutrition			
	*N*	Mean	SD	*N*	Mean	SD	*N*	Mean	SD	*p* Value *	*N*	Mean	SD	*N*	Mean	SD	*N*	Mean	SD	*p* Value *
**HADS**																				
Total score anxiety	260	4.98	4.04	116	4.79	3.66	144	5.13	4.33	0.889	176	3.36	3.70	79	3.27	3.38	97	3.44	3.96	0.872
Total score depression	261	2.69	3.02	116	2.58	2.84	145	2.78	3.16	0.948	179	2.55	3.13	80	2.39	3.09	99	2.68	3.17	0.489
**EORTC QLQ-H&N35 ^a^**																				
Pain	263	21.05	21.66	116	25.36	22.61	147	17.65	20.31	**0.003**	181	14.61	18.01	81	18.66	19.85	100	11.33	15.87	**0.009**
Swallowing	262	13.01	21.36	116	17.81	23.13	146	9.19	19.08	**<0.001**	180	13.80	18.86	80	18.85	21.08	100	9.75	15.85	**<0.001**
Senses problems	263	10.27	21.21	116	14.66	26.11	147	6.80	15.58	**0.013**	181	23.57	25.76	81	27.37	26.91	100	20.50	24.49	0.051
Speech problems	260	14.15	19.03	115	13.72	18.58	145	14.48	19.44	0.797	177	12.99	18.63	80	14.58	20.28	97	11.68	17.15	0.319
Trouble social eating	258	13.86	19.66	114	18.06	21.72	144	10.53	17.21	**<0.001**	176	15.47	19.03	79	18.95	19.56	97	12.63	18.21	**0.007**
Trouble social contact	260	5.35	12.02	115	3.70	8.71	145	6.67	13.99	0.108	177	4.81	10.47	80	4.11	8.64	97	5.38	11.77	0.710
Less sexuality	244	27.46	33.53	108	28.24	32.66	136	26.84	34.32	0.540	170	24.12	31.35	76	22.59	30.03	94	25.35	32.49	0.577
Teeth	261	13.15	25.35	116	12.64	25.12	145	13.56	25.61	0.731	179	17.50	25.80	81	18.93	26.32	98	16.33	25.44	0.458
Opening mouth	263	12.17	24.45	116	14.37	24.95	147	10.43	23.99	0.057	180	13.33	25.55	81	15.23	24.75	99	11.78	26.22	0.120
Dry mouth	263	23.07	29.85	116	21.55	29.57	147	24.26	30.11	0.377	180	48.52	33.30	81	55.97	31.55	99	42.42	33.61	**0.005**
Sticky saliva	263	20.91	28.65	116	22.70	29.36	147	19.50	28.09	0.325	179	35.01	32.44	80	41.25	33.23	99	29.97	31.04	**0.017**
Coughing	263	20.41	25.92	116	22.13	26.36	147	19.05	25.58	0.283	181	20.99	26.55	81	21.40	24.33	100	20.67	28.34	0.464
Feeling ill	262	16.67	24.89	116	17.82	24.65	146	15.75	25.13	0.371	178	11.61	21.32	79	12.24	20.10	99	11.11	22.34	0.463
*Pain killers*	260	53.85	49.95	115	61.74	48.82	145	47.59	50.12	**0.023**	177	33.90	47.47	80	37.50	48.72	97	30.93	46.46	0.359
*Nutritional supplements*	260	18.46	38.87	115	26.96	44.57	145	11.72	32.28	**0.002**	177	19.21	39.51	80	18.75	39.28	97	19.59	39.89	0.888
*Feeding tube*	260	3.08	17.30	115	4.35	20.48	145	2.07	14.28	0.292	176	3.98	19.60	80	6.25	24.36	96	2.08	14.36	0.160
*Weight loss*	259	27.80	44.89	114	38.60	48.90	145	19.31	39.61	**0.001**	176	16.48	37.20	80	17.50	38.24	96	15.63	36.50	0.739
*Weight gain*	256	12.89	33.58	114	14.04	34.89	142	11.97	32.58	0.625	176	32.39	46.93	80	33.75	47.58	96	31.25	46.60	0.725

*HADS* Hospital Anxiety and Depression Scale, *EORTC QLQ-H&N35* European Organization for Research and Treatment of Cancer Quality of Life Questionnaire-Head and Neck 35. ^a^ Higher scores indicate more severe symptoms. For parameters in italics, the scores indicate the percentage of yes answers. * Malnutrition according to GLIM vs. no malnutrition using the Mann–Whitney U-test. Statistically significant *p*-values (<0.05) are shown in bold text.

## Appendix B

**Table A2:** Clinically relevant deterioration from the start of treatment to one year after the termination of treatment in a number of EORTC QLQ-H&N35 scales proven to be significantly different between patients with malnutrition defined by the Global Leadership Initiative on Malnutrition (GLIM) and patients without malnutrition.

	Malnutrition			No Malnutrition		
	n	S ≥ 10 *	%	n	S ≥ 10 *	%
Dry mouth	80	56	70.0	97	49	50.5
Sticky saliva	79	41	51.9	97	43	44.3
Trouble with social eating	77	23	29.9	93	23	24.7
Swallowing	79	21	26.6	97	18	18.6
Pain	80	13	16.3	98	15	15.3

* S ≥ 10: Number of patients reporting scores from the EORTC QLQ-H&N35 European Organization for Research and Treatment of Cancer Quality of Life Questionnaire-Head and Neck 35 at one year after the termination of treatment of at least 10 points higher compared to the score reported at the start of treatment, indicating a clinically relevant deterioration of the symptom.
